# Management of persistent caesarean scar pregnancy after curettage treatment failure

**DOI:** 10.1186/s12884-017-1395-4

**Published:** 2017-07-01

**Authors:** Zhi-Da Qian, Yue Weng, Yong-Jiang Du, Chun-Fen Wang, Li-Li Huang

**Affiliations:** 1 Women’s Hospital, School of Medicine, Zhejiang University, 1 Xueshi Road, Hangzhou, Zhejiang Province 310006 People’s Republic of China; 2 Maternal and Child Health Institute of Lin’an City, 25 Jiangnan Road, Lin’an, Zhejiang Province 311300 People’s Republic of China

**Keywords:** Caesarean scar pregnancy, Dilatation and curettage, Hysteroscopy, Uterine artery embolisation, Treatment

## Abstract

**Background:**

Caesarean scar pregnancy (CSP) is a late serious complication of caesarean section. The incidence of CSP has increased worldwide in recent years. Early diagnosis and prompt therapy are crucial to avoid catastrophic complications. There are various strategies for CSP treatment, but there is no consensus on the best management for CSP. Dilation and curettage (D&C) and hysteroscopy are common and effective treatments with their advantages and disadvantages. No in-depth study of the clinical effects of hysteroscopic management of CSP after D&C treatment failure has been conducted. The purpose of this study is to evaluate the effectiveness and safety of hysteroscopic removal of residual CSP tissue (persistent CSP) as a rescue after failed D&C management.

**Methods:**

This is a retrospective clinical research study. Forty-five patients underwent operative hysteroscopy to remove the residual gestational tissue in the caesarean scar after failed D&C treatment. The clinical characteristics and outcomes of hysteroscopic surgeries of 45 CSP cases were investigated**.** All data analyses were conducted with SPSS 17.0.

**Results:**

Forty-three CSP cases after unsuccessful curettage treatment were successfully treated by operative hysteroscopy. The estimated intraoperative blood loss was 20.00 (10.00–500.00) mL, the hysteroscopic operating time was 20.00 (15.00–45.00) min, the decline of serum *β*-hCG the day after surgery was 71.91 ± 14.05%, the total hospitalisation time was 7.87 ± 2.26 days, the medical cost was 13,682.71 ± 3553.77 China Yuan (CNY), the time of bleeding after surgery was 7.42 ± 2.48 days, and the time of serum *β*-hCG resolution after surgery was 13.84 ± 9.83 days. Follow-up after discharge demonstrated that there were no severe complications for any patients.

**Conclusions:**

Hysteroscopy therapy could treat persistent CSP effectively and safely after curettage treatment failure. Therapy should be individualised, and the risks and cost of the hysteroscopy procedure and anaesthesia must be considered and fully discussed with the patients before surgery.

## Background

A caesarean scar pregnancy (CSP) is a late serious complication of caesarean section (CS). CSP is defined as a gestational sac located in the scar of an earlier caesarean delivery. As a special form of ectopic pregnancy, CSP was first reported by Larsen and Solomon in 1978 [1]. The incidence of CSP is one in 1800–2216 pregnancies [2]. Prior CS represents the most common predisposing factor for CSP [3]. There has been a rapid increase in the incidence of CSP worldwide, especially in China in recent years. This increased incidence may have contributed to the in-depth understanding of CSP, the high CS rate, and the more wide-spread use of colour Doppler sonography [4, 5]. The phasing out of the one child policy in China has also contributed to the increasing incidence of CSP. If such a pregnancy is allowed to continue, the patient is at risk for uterine scar rupture with haemorrhage (even in the first trimester), hysterectomy, and loss of sequent fertility.

Accurate diagnosis and prompt therapy are important to avoid catastrophic complications. There are various strategies for CSP therapy, and the uterine conserving termination of CSP (medical or surgical) in the first trimester has been strongly preferred [2]. As less-invasive management options, a variety of medications (methotrexate (MTX), etoposide, and potassium chloride) have been adopted in the treatment of CSP [2, 4, 6]. Surgical managements include dilation and curettage (D&C) [7–9], uterine artery embolisation (UAE) [10] coupled with MTX arterial injection [11], hysteroscopy [12–15], and laparoscopy [16]. Hysterectomy is usually performed in emergencies such as heavy bleeding or uterine rupture. It is hard to decide on the optimal management for individual CSP cases.

As a traditional and primary treatment of CSP [7–9], D&C under transabdominal sonography (TAS) guidance after preventive UAE is simple and effective in most cases [5, 10]. D&C carries low cost and good curative effect, but it is accompanied by risks, including sever haemorrhage, low success rate, and even uterine perforation due to lack of direct visualisation. It often takes a longer time for the normalisation of the serum beta-human chorionic gonadotropin (*β*-hCG) level and resolution of the CSP mass. Some patients require further treatments and even hysterectomy [17, 18]. As reported, for CSP patients who had continuous vaginal bleeding and abnormal serum levels of *β*-HCG after medication and D&C, the remaining ectopic gestational tissue could be successfully hysteroscopically removed [15, 19, 20]. According to recent small case series, hysteroscopic removal of CSP is an alternative minimally invasive operation that offers direct visualisation, definitive confirmation of the diagnosis, removal of the gestational tissue entirely, short operative time and postoperative stay, and rapid normalisation of *β*-hCG. Although it is effective and safe, it requires experienced surgeons who are familiar with the anatomy of the uterine cavity and who are skilful at manipulating hysteroscopic instruments to prevent heavy bleeding and to keep the operative field clear. Facilities for immediate laparotomy or laparoscopy must be available. As a minimally invasive treatment, UAE is alternatively used to prevent and control severe haemorrhage and to preserve the patient’s uterus and future fertility. Women with CSP who experienced preventive UAE before surgery have a high success rate and a low complication rate [21].

Although they have been reported with success, both D&C and hysteroscopy treatments have their advantages and disadvantages. Furthermore, most of literature reports were small case series or case reports. There are no in-depth studies of the clinical effects of hysteroscopic treatment of persistent CSP after D&C management failure. We conducted this retrospective clinical research to evaluate the clinical outcomes of hysteroscopic removal of residual gestational tissue as a rescue in the treatment of CSP patients with failed D&C. We found that the operative hysteroscopy was safe and effective.

## Methods

This retrospective clinical investigation was conducted in the Department of Obstetrics and Gynaecology, Women’s Hospital, School of Medicine, Zhejiang University, People’s Republic of China, between March 2011 and September 2014. This hospital is the largest tertiary obstetrics and gynaecology medical treatment centre in Zhejiang province. Informed consent for the surgery and participation was obtained from each patient in the study, and the protocol was approved by the Institutional Review Board in the Women’s Hospital, School of Medicine, Zhejiang University.

### Subjects’ criteria

Women with CSP who underwent hysteroscopic management after D&C were enrolled in the study if they were ≤12 weeks of gestation (at the time of D&C), haemodynamically stable without internal bleeding, and had an unruptured type of CSP. The actual gestational age was calculated based on last menstrual period and adjusted according to the date of the positive early pregnancy test, the ultrasound examination, the appearance of morning sickness, and whether the patient’s menstruation was regular or not. Exclusion criteria included cervical pregnancy, inevitable abortion, incomplete abortion, missed abortion, caesarean scar choriocarcinoma, and significant maternal hepatic, renal, cardiac or blood system disease.

### Diagnosis standard of CSP

**Fig. 1:**
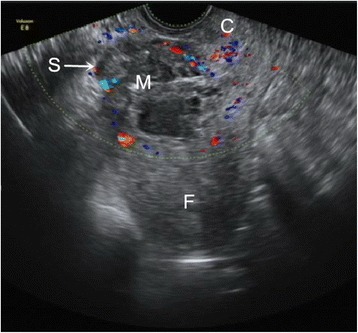
Transvaginal ultrasonography of the patient with caesarean scar pregnancy after curettage treatment failure. The uterine cavity and cervical canal were empty. A 5.4 × 3.7 × 3.7 cm mass implanted in the anterior wall of the uterine caesarean scar (*arrow*) embedded and surrounded by thin myometrium and separated from the endometrial cavity was visible on the retroverted uterus. Ultrasonography revealed that the mass was heterogeneous with a mixture of cystic and solid echogenicity. C = cervix; F = fundal endometrial cavity; M = mass; S = scar of caesarean section

The CSP was diagnosed using the following criteria: a history of low-transverse caesarean delivery in the lower uterine segment, positive urine pregnancy test or serum *β*-HCG level, and fulfilment of the following ultrasonography criteria [22] (Fig. 1): (a) Development of the gestational sac in the anterior portion of the lower uterine segment; (b) Empty uterine cavity and cervical canal; and (c) Absence of healthy myometrium between the gestational sac and the bladder. Magnetic resonance imaging (MRI) and three-dimensional (3-D) power Doppler ultrasound might be useful in the cases in which the diagnosis remains unclear after transvaginal sonography (TVS) examination.

### Management

Patients were readmitted due to intermittent vaginal bleeding, severe bleeding, a prolonged return of *β*-hCG to normal, or the persistence of a caesarean scar mass after D&C. All patients were initially treated with a D&C regimen only or D&C combined with preventive UAE. They were counselled extensively regarding the abnormal vaginal bleeding, the abnormal *β*-hCG levels, the residual gestational tissue, and the further treatment indications. With the diagnosis of persistent CSP, medical and surgical options (including administration of MTX, hysteroscopic resection, and laparoscopy or laparotomy resection) were discussed with them before they were treated with operative hysteroscopy combined with or without UAE.

Super selective embolisation of both uterine arteries was performed using gelatine sponge powder by two experienced radiologists. Under local anaesthesia, the Seldinger technique was applied to puncture and catheterize via the right femoral artery, into the bilateral internal iliac arteries to perform pelvic artery digital subtraction angiography. Post UAE angiography was performed to confirm that the vessel occlusion was complete.

**Fig. 2:**
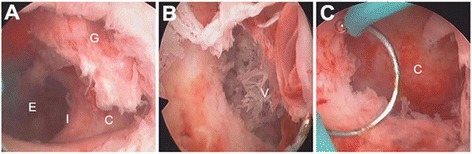
Hysteroscopic view of the caesarean scar pregnancy. C = caesarean scar diverticulum; E = endometrial cavity; G = gestational tissue; I = internal os; V = villous. **a** Before operation: the endometrial cavity was empty, and the gestational tissue was implanted in a diverticulum located in the left anterior endocervical wall, compatible with prior caesarean section scar. **b** During operation: the residual villous in a niche located in the left endocervical wall (disruption of the caesarean scar). **c** After operation: the residual gestational tissue was removed completely, and there was no obvious bleeding point

All patients underwent careful hysteroscopic surgeries (Fig. 2) by experienced gynaecologists to remove the products of conception under TAS (LOGIQ P6, GE, USA) guidance. The cervical dilatation was carefully and successively accomplished by Hegar dilators from 5 mm to 10.5 mm. Under epidural anaesthesia, an operative hysteroscope (CA95138, Stryker, USA) with a 10-mm external diameter was placed inside the uterus. A continuous flow 26F hysteroscopic resectoscope with a 90-degree wire loop electrode was used under the guidance of TAS. Propelled by a uterine expansion instrument, 5% glucose solution was used to obtain uterine distension. An electrosurgical generator (VIO300S, ERBE, Germany) was used on a setting of 70 W of coagulation current and 70 W of cutting current, respectively.

Hysteroscopy recognised the implantation of the persistent ectopic gestational tissue under direct visualisation. The vessel bed of the implantation site was exposed and coagulated for haemostasis first. If the diameter of residual gestational tissue was more than 2 cm, an oval pair of forceps was used to remove it under TAS guidance. Then, the electric loop was used carefully without electricity to curettage the residual gestational tissue on the wall of the diverticulum (disruption of the caesarean scar) to decrease the risk of uterine scar rupture and to remove the residual villous. When the residual gestational tissue was closely attached to the uterus wall, the electric cutting was used to ensure the remaining tissue was removed completely. Finally, the uterine scar diverticulum was checked thoroughly to confirm that there were no obvious residual tissue and active bleeding.

The amount of bleeding during surgery was observed in all patients. Iodoform gauze packing was left in situ for 24–48 h when there was heavy bleeding (800–1500 mL). Hysterectomy was performed immediately if the blood loss was more than 1500 mL. The serum *β*-hCG level, liver and kidney function tests, and routine blood tests were examined before surgery, the day after surgery, and 7 days after surgery. The size of the residual mass was measured by TVS at the same time.

### Investigation content

All patients were observed during the hospitalisation. The clinical characteristics of 45 CSP patients were investigated, and the variables included maternal age, gravidity, number of prior CSs, number of abortions, interval between CSP and CS, gestational age, interval between hysteroscopic surgery and D&C, mean size of the residual gestational tissue before hysteroscopic surgery, thickness of the lower uterine segment, estimated intraoperative blood loss, hysteroscopic operating time, serum *β*-hCG levels before and after hysteroscopic surgery, side effects (such as haemorrhagic shock and infection), number of hysterectomy, medical cost, hospitalisation time after surgery, and total hospitalisation time.

All patients were retrospectively investigated more than 1 year after hysteroscopic surgeries. Their serum *β*-hCG levels were followed-up weekly until the normalisation. At half a month, 1 month (2 months or 3 months, if needed) postoperation, TVS was performed to evaluate the recovery of the uterus. Other information about the 45 patients were investigated, including their clinical status (lower abdominal pain, bleeding pattern, or any other symptoms), days for *β*-hCG to return to normal (<5.3 IU/L), and the time of vaginal bleeding after surgery.

### Statistical analysis

All the data were analysed by SPSS 17.0 software (SPSS, Inc., USA) for homogeneity of variance with the Levene test and for normal distribution with the Kolmogorov–Smirnov test. If variables met assumptions of homogeneity of variance and normality, the independent samples *t* test was used (mean ± SD), If not, the nonparametric test (the Mann–Whitney *U* test) was used [median (range)].

## Results

A total of 1348 women were diagnosed as CSP in our hospital during the study period, and 154 (11.42%) of them were readmitted due to retained products of conception or other symptoms. Forty-five (3.34%) CSP patients underwent operative hysteroscopy after the initial treatment of D&C (41 patients underwent D&C regimen only, and 4 patients underwent D&C combined with preventive UAE) during the study period. They were readmitted due to the persistence of caesarean scar mass (45/45) accompanied by intermittent vaginal bleeding (21/45), severe bleeding (7/45), and a prolonged return time of *β*-hCG to normal (9/45). With the diagnosis of persistent CSP, they were treated with operative hysteroscopy only (10/45) or in combination with UAE (35/45). In all patients, histopathologic examination performed on residual tissue obtained at hysteroscopic surgery confirmed the diagnosis of persistent CSP.

The patients’ characteristics and demographics (Table 1): the maternal age was 32.84 ± 4.37 years old, the gravidity was 4.49 ± 1.41 times, the number of prior abortions was 2.00 (0.00–6.00) times, the number of prior CSs was 1.00 (1.00–2.00) times, the interval between CS and CSP was 71.44 ± 46.17 months, the gestational age was 83.18 ± 28.14 days, the interval between D&C and hysteroscopic surgery was 22.33 ± 13.08 days, the serum *β*-hCG level before hysteroscopic surgery was 2623.00 (0.55–39,992.00) IU/L, the size of the residual gestational tissue before hysteroscopic surgery was 3.44 ± 1.12 cm, and the thickness of the lower uterine segment was 1.20 (0.50–8.00) mm.

**Table 1:** The patients’ characteristics and demographics

Characteristic	Value
Maternal age (years)	32.84 ± 4.37
Gravidity (times)	4.49 ± 1.41
Prior abortion (times)	2.00 (0.00–6.00)^a^
Prior CS (times)	1.00 (1.00–2.00)^a^
Interval between CS and CSP (months)	71.44 ± 46.17
Gestational age (days)	83.18 ± 28.14
Interval between D&C and hysteroscopic surgery (days)	22.33 ± 13.08
Serum *β*-hCG level before hysteroscopic surgery (IU/L)	2623.00 (0.55–39,992.00)^a^
Size of the residual gestational tissue before hysteroscopic surgery (cm)	3.44 ± 1.12
Thickness of the lower uterine segment (mm)	1.20 (0.50–8.00)^a^

Normal distribution (mean ± SD), Non-normal distribution (median [range]). Unless noted ^a^otherwise, values are presented as mean ± SD

*CS* cesarean section, *CSP* cesarean scar pregnancy, *D&C* dilatation and curettage

The outcomes of hysteroscopic surgeries (Table 2): the estimated intraoperative blood loss was 20.00 (10.00–500.00) mL, the hysteroscopic operating time was 20.00 (15.00–45.00) min, the serum *β*-hCG level the day after hysteroscopic surgery was 675.80 (0.20–16,336.00) IU/L, the decline of serum *β*-hCG the day after surgery was 71.91 ± 14.05%, the medical cost was 13,682.71 ± 3553.77 China Yuan (CNY), the hospitalisation time after hysteroscopic surgery was 4.47 ± 1.83 days, the total hospitalisation time was 7.87 ± 2.26 days, the time of bleeding after surgery was 7.42 ± 2.48 days, and the time of serum *β*-hCG resolution after surgery was 13.84 ± 9.83 days. Follow-up after discharge showed that there were no severe complications for any women.

**Table 2:** Outcomes of hysteroscopic surgeries

Characteristic	Value
Success rate (%)	95.56
Side effect (%)	8.89
Estimated intraoperative blood loss (mL)	20.00 (10.00–500.00)^a^
Hysteroscopic operating time (min)	20.00 (15.00–45.00)^a^
Serum *β*-hCG level the day after hysteroscopic surgery (IU/L)	675.80 (0.20–16,336.00)^a^
Decline of serum *β*-hCG the day after surgery (%)	71.91 ± 14.05
Total hospitalization time (days)	7.87 ± 2.26
Hospitalization time after hysteroscopic surgery (days)	4.47 ± 1.83
Medical cost (CNY)	13,682.71 ± 3553.77
Time of bleeding after surgery (days)	7.42 ± 2.48
Time of serum *β*-hCG resolution after surgery (days)	13.84 ± 9.83

Normal distribution (mean ± SD), Non-normal distribution (median [range]). Unless noted ^a^otherwise, values are presented as mean ± SD

*CS* cesarean section, *CSP* cesarean scar pregnancy, *CNY* China Yuan

The success rate was 95.56% (43/45) (definition of success: patient needs no more operations or medication therapy during or after hysteroscopic surgery, and her fertility is preserved), and no patient underwent hysterectomy. A 30-year-old woman was treated with a single dose of intramuscular MTX (50 mg/m^2^ body surface area) and mifepristone (50 mg twice a day for 3 days) 4 days after hysteroscopic operation combined with UAE owing to a serum *β*-hCG level that was decreased less than 50% from the preoperative level, and it decreased to normal 21 days after surgery. Another 35-year-old woman was offered operative hysteroscopy without preventive UAE. Emergency UAE was performed successfully after unsuccessful iodoform gauze tamponade in this patient owing to haemorrhage (500 mL) during hysteroscopic surgery. There were no other severe complications for this patient.

The side effect rate was 8.89% (4/45), and there were no severe side effects in the research. One patient had a pelvic infection after hysteroscopy. She was a 36-year-old woman, and she had a history of irregular vaginal bleeding for 131 days at the hysteroscopic operation. On the first day after surgery, she started to have fever (38.7 °C) accompany with a mild lower abdominal pain. Bimanual examination revealed uterine tenderness, and motion of the cervix and uterus caused increased pain. Intravenous antibiotics were continued until the patient had been afebrile for 48 h. The patient was discharged 9 days after operation. There were 2 patients who suffered mild abnormalities of liver function after hysteroscopy combined with UAE. Their liver functions were normal a few days after the liver-protecting treatment. Another patient had experienced chest congestion at the end of hysteroscopy, and her vital signs were stable at that time. The symptom disappeared soon after the procedure was stopped immediately. The other 41 patients were discharged without any side effects and complications.

The hysteroscopy revealed a large caesarean scar defect (diverticulum) embedded with residual gestational tissues and an empty uterine cavity (Fig. 2). Thirteen patients with intrauterine adhesions were diagnosed during the hysteroscopy procedure and were simultaneously treated with hysteroscopic adhesiolysis.

## Discussion

As a rare type of ectopic pregnancy, it is crucial to make an accurate diagnosis and to provide prompt therapy to avoid catastrophic complications. According to earlier reports, expectant management is not recommended owing to a low success rate and poor prognosis [3, 23]. It is generally recommended to terminate CSP in the first trimester as soon as possible after diagnosis. Haemodynamically stable cases have more conservative management options.

Although D&C is used as a traditional and simple treatment of CSP, it often coupled with a high rate of haemorrhage even when sonographically guided [6] and is accompanied by prolonged resolution time of *β*-hCG levels and of the CSP mass. Other problems might not be acceptable for doctors and patients, such as extended surveillance time and intermittent vaginal bleeding, and some patients require further management. Sadeghi et al. [18] reported that 8.33% of CSP patients initially treated with D&C ultimately required hysterectomy.

It is controversial whether intervention is clinically justified based on a slow decline in *β*-hCG level or ultrasound evidence of retained tissue. Jurkovic et al. [24] reported that 15% of CSP patients attending for follow-up had ultrasound evidence of retained products of conception, but only 6% required repeat intervention based on their clinical symptoms. They did not use *β*-hCG for follow up. In this study, 11.42% (154/1348) CSP patients were readmitted due to a persistent caesarean scar mass, abnormal *β*-hCG level, and other symptoms. Only 45 (3.34%) underwent operative hysteroscopy to remove the residual gestational tissue due to the persistence of caesarean scar mass (45/45) accompanied by intermittent vaginal bleeding (21/45), severe bleeding (7/45), and a prolonged return time of *β*-hCG to normal (9/45). The other 8 patients only had ultrasound evidence of retained tissue. These 17 asymptomatic patients were so worried about their retained tissue and the slow decline in *β*-hCG level that they underwent hysteroscopy surgeries after full discussion and informed consent. It is important to follow-up with patients after D&C management to identify evidence of persistent CSP. The surgical indication for persistent CSP is contentious, and it is valuable to carry out further research to reach a more definitive conclusion.

Further management of persistent CSP after D&C treatment failure includes expectant management, systemic or local MTX, repeat curettage, hysteroscopy, laparoscopy, laparotomy, and hysterectomy. MTX treatment is a less-invasive and appropriate management option when the woman is clinically stable and has an unruptured CSP [6]. However, it might also be associated with nausea, stomatitis, impairment of liver and renal function, abnormal vaginal spotting or even severe bleeding, and a prolonged return time of *β*-hCG. Due to the lack of direct visualisation, repeat curettage under ultrasound guidance carries the possibility of residue, haemorrhage, uterine perforation, and low success rate. Laparotomy and laparoscopy could remove the residual gestational tissue and repair the uterine defect (or ‘niche’) simultaneously. Ben et al. [25] reported a CSP case that underwent successful repair of the uterine defect, resulting in subsequent normal intrauterine pregnancy. However, abdominal surgery and uterine repair might be complicated by postoperative intra-abdominal adhesions and poor scar healing, both of which might have adverse effects on women’s future fertility. Hysterectomy is usually performed in emergency situations (heavy bleeding or uterus rupture) in the treatment of persistent CSP.

Hysteroscopic removal of CSP might be used as an effective rescue after D&C or medical management failure [15, 19, 20]. It offers an accurate diagnosis by observing the gestational tissue at the implantation site. Operative hysteroscopy also offers appropriate management by separating the gestational tissue from the uterine wall and coagulating the blood vessels directly. Deans et al. [15] and Chao et al [19] reported that hysteroscopy is an important alternative method for CSP with less blood loss, shorter operating time, and rapid normalisation of the *β*-hCG level. We retrospectively investigated the use of TAS guided operative hysteroscopy to manage persistent CSP after unsuccessful D&C treatment. Our findings were similar with their studies. Laparoscopically assisted operative hysteroscopy has the additional advantages of minimally invasive caesarean scar resection, rapid and effective management of haemorrhage, and immediate detection and address uterine and/or bladder perforation [26, 27].

We found that the readmission reasons included the persistence of caesarean scar mass, abnormal vaginal bleeding, and prolonged decrease of *β*-hCG. It suggests that whatever treatment is selected, follow-up of symptoms, caesarean scar mass, and *β*-hCG level are necessary to ensure complete resolution of CSP. When surgical removal is performed in ectopic pregnancy without residual chorionic villi, the serum *β*-hCG level should be no more than 1/2 of its pre-surgical level at 24 h after operation and should continually decrease 1/2 for each subsequent 24 h [28]. If the serum *β*-hCG level falls less than that, it suggests the possibility of trophoblastic tissue remaining (persistent CSP) [26]. In this study, the curve of the serum *β*-hCG level decline after D&C in some patients is less dramatic due to the residual gestational tissue. It seems that simple suction evacuation and curettage would frequently leave chorionic villi imbedded within the caesarean scar defect.

Preventive UAE followed by curettage or hysteroscopy is an alternative, effective, safe, and minimally invasive treatment for CSP [10, 21]. The purpose of UAE before surgery includes blocking the blood flow in uterine arteries, decreasing local vascularisation, and inducing trophoblastic degeneration. Patients treated with UAE before D&C have less intraoperative blood loss, shorter hospitalisation time, lower rate of hysterectomy, quicker normalisation of serum *β*-hCG level, and shorter recovery time than those without UAE. However, there were only 4 cases who underwent preventive UAE before D&C in this study, and this low rate might be related to persistent CSP. From our experience, preventive UAE before D&C should be recommended in cases with ≥8 weeks of gestation, a CSP mass diameter ≥ 6 cm, a lesion bulging into the uterovesical fold, and abundant blood flow signals around the lesion under pulsed Doppler ultrasonography. In this report, 35 patients were treated with operative hysteroscopy combined with UAE. The performance of hysteroscopy therapy treating the persistent CSP effectively and safely might be partly owed to the fact that most patients underwent preventive UAE before operative hysteroscopy to prevent massive bleeding.

There were two patients who failed in this study. One case was treated with mifepristone and MTX a few days after hysteroscopy owing to a slow decrease in serum *β*-hCG level. The hysteroscopy revealed a large caesarean scar defect in the left endocervical wall deeply embedded with gestational tissue by direct visualisation. However, it was hard to completely remove the residual tissue in the niche due to the special position and villous implantation. She was diagnosed as persistent CSP after hysteroscopy and was offered medication therapy. Another patient underwent emergency UAE owing to massive bleeding during hysteroscopic surgery. This complication might be due to her big residual gestational mass with abundant blood flow and a thinner myometrium at the implantation site. The presence of a large CS scar defect might lead to haemorrhage because of weakened myometrial contractility. It is hard to prevent severe haemorrhage under hysteroscopy in this situation because active bleeding could impair vision and block the operative field. In the case of heavy bleeding after the residual gestational tissue is removed, Foley tamponade or intrauterine gauze compression might be tried initially. When other management techniques have failed to stop the bleeding, hysterectomy is performed as the last step to save the patient’s life. We recommend that the selection of specific surgical procedures should be according to the size of the CSP mass and its implantation site. Laparoscopy or laparoscopically assisted operative hysteroscopy might be more suitable for a patient with a large CSP mass deeply implanted into a CS defect, especially for a patient with the residual tissue bulge into the uterovesical fold under the caesarean scar.

One patient suffered chest congestion during hysteroscopic surgery, suggesting that cases treated by operative hysteroscopy might have additional risks from anaesthesia and from the hysteroscopy procedure. Although hysteroscopy is considered a safe procedure and is used widely, complications such as cervical laceration, uterine perforation, haemorrhage, absorption of irrigation solutions (water intoxication), and air embolism might occur. Although epidural anaesthesia is safe and effective, some complications might occur, including internal bleeding, epidural haematoma, nerve injury, mucosa or skin oedema, anaesthetic toxicity reaction, and coma. To detect mismanagement and limit the extent of injury, careful monitoring during the hysteroscopy procedure and epidural blocking should be taken. The hysteroscopy hospitalisation cost might be significantly higher than D&C management only. The additional financial burden and risks owing to hysteroscopy and anaesthesia must be considered and carefully discussed with the patients before operation.

## Conclusions

A persistent CSP after unsuccessful D&C treatment could be effectively treated by operative hysteroscopy with or without UAE. However, hysteroscopic surgery has additional risks and costs owing to the anaesthesia and hysteroscopy procedures. For an inexperienced surgeon, operative hysteroscopic management of CSP might be very difficult. There is no agreement on the optimum management of CSP; treatment should be individualised, and some conditions must be carefully considered and discussed before making a therapy plan with the patient, including the patient’s age, history of gestation, haemodynamic stability, size of gestational tissue, serum *β*-hCG level, and desire for future fertility. Whatever treatment is selected, follow-up with symptoms, serum *β*-hCG level, and uterus ultrasound are necessary to ensure complete resolution of the pregnancy. It is important to carry out high-quality, large-sample, longer follow-up trials to reach more definitive conclusions.

## Abbreviations

CNY: China Yuan
CS: Caesarean section
CSP: Caesarean scar pregnancy
D&C: Dilation and curettage
MRI: Magnetic resonance imaging
MTX: Methotrexate
TAS: Transabdominal sonography
TVS: transvaginal sonography
UAE: Uterine artery embolisation
*β*-HCG: Beta-human chorionic gonadotropin
